# Intestinal microbiota drives cholestasis-induced specific hepatic gene expression patterns

**DOI:** 10.1080/19490976.2021.1911534

**Published:** 2021-04-13

**Authors:** Oriol Juanola, Mohsin Hassan, Pavitra Kumar, Bahtiyar Yilmaz, Irene Keller, Cédric Simillion, Cornelius Engelmann, Frank Tacke, Jean-François Dufour, Andrea De Gottardi, Sheida Moghadamrad

**Affiliations:** a Translational Research Laboratory, Gastroenterology and Hepatology, Ente Ospedaliero Cantonale, Università Della Svizzera Italiana, Lugano, Switzerland; b Department of Hepatology & Gastroenterology, Charité Universitätsmedizin Berlin, Berlin, Germany; c Hepatology, Department for Biomedical Research, University of Bern, Bern, Switzerland; d Gastroenterology, Department for Biomedical Research, University of Bern, Bern, Switzerland; e University Clinic of Visceral Surgery and Medicine, Inselspital, Bern, Switzerland; f Department for Biomedical Research and Swiss Institute of Bioinformatics, University of Bern, Bern, Switzerland; g Institute for Liver and Digestive Health, University College London, London, UK; h Berlin Institute of Health (BIH), Berlin, Germany

**Keywords:** Intestinal microbiota, acute cholestasis, bile acids, gene expression, germ-free mice, metabolism

## Abstract

Intestinal microbiota regulates multiple host metabolic and immunological processes. Consequently, any difference in its qualitative and quantitative composition is susceptible to exert significant effects, in particular along the gut-liver axis. Indeed, recent findings suggest that such changes modulate the severity and the evolution of a wide spectrum of hepatobiliary disorders. However, the mechanisms linking intestinal microbiota and the pathogenesis of liver disease remain largely unknown. In this work, we investigated how a distinct composition of the intestinal microbiota, in comparison with germ-free conditions, may lead to different outcomes in an experimental model of acute cholestasis. Acute cholestasis was induced in germ-free (GF) and altered Schaedler’s flora (ASF) colonized mice by common bile duct ligation (BDL). Studies were performed 5 days after BDL and hepatic histology, gene expression, inflammation, lipids metabolism, and mitochondrial functioning were evaluated in normal and cholestatic mice. Differences in plasma concentration of bile acids (BA) were evaluated by UHPLC-HRMS. The absence of intestinal microbiota was associated with significant aggravation of hepatic bile infarcts after BDL. At baseline, we found the absence of gut microbiota induced altered expression of genes involved in the metabolism of fatty and amino acids. In contrast, acute cholestasis induced altered expression of genes associated with extracellular matrix, cell cycle, autophagy, activation of MAPK, inflammation, metabolism of lipids, and mitochondrial functioning pathways. Ductular reactions, cell proliferation, deposition of collagen 1 and autophagy were increased in the presence of microbiota after BDL whereas GF mice were more susceptible to hepatic inflammation as evidenced by increased gene expression levels of osteopontin, interleukin (IL)-1β and activation of the ERK/MAPK pathway as compared to ASF colonized mice. Additonally, we found that the presence of microbiota provided partial protection to the mitochondrial functioning and impairment in the fatty acid metabolism after BDL. The concentration of the majority of BA markedly increased after BDL in both groups without remarkable differences according to the hygiene status of the mice. In conclusion, acute cholestasis induced more severe liver injury in GF mice compared to mice with limited intestinal bacterial colonization. This protective effect was associated with different hepatic gene expression profiles mostly related to tissue repair, metabolic and immune functions. Our findings suggest that microbial-induced differences may impact the course of cholestasis and modulate liver injury, offering a background for novel therapies based on the modulation of the intestinal microbiota.

## Introduction

The mammalian gut harbors billions of microorganisms, including Archaea and Eukaryota, collectively referred as gut microbiota. There is increasing evidence that humans have evolved a close symbiotic relationship with gut microbiota. An individual variation in the gut microbial species may implicate several diseases. Over the last few decades a number of studies have shown that the gut microbiota produces a large repertoire of small molecules and contributes to important functions of host physiology including immunity and metabolism,^1–4^ digestive^4^ and protective functions,^5^ microbial catabolism of otherwise indigestible nutrients,^6^ provision of essential amino acids, maturation of the host mucosal immune system,^5,7^ completion of the bile-salt cycle and pre-systemic metabolism of drugs and toxins.^8–10^ The bidirectional interaction between the gut microbiota and the liver is established through a vascular route consisting of the portal vein that carries gut-derived metabolites to the liver, and a biliary route, that transports the bile from liver to the intestine.^11^ Indeed, changes in intestinal bacterial composition and the derived metabolites play a key role in the pathogenesis of a number of chronic diseases including liver disorders.^12,13^ The alteration in gut microbial composition and its subsequent effect on different metabolic disorders have been recently highlighted in numerous experimental models.^14,15^ For example, a study by Rabot *et al*. compared the metabolic consequences of the absence of intestinal microbiota in mice treated with a high fat diet. Germ-free (GF) mice consumed less calories, excreted more fecal lipids and consequently their body weight was significantly lower than colonized mice, emphasizing the direct effect of microbiota on energy metabolism.^16^ Studies have also demonstrated that intestinal microbiota can influence the total energy balance equation and genes regulating energy expenditure.^15,17^ Moreover, the absence of gut microbiota has been associated with a reduced BA diversity,^18,19^ increased size of the BA pool, the absence of secondary BA, reduced fecal excretion of BA and up-regulation of BA transporter genes.^20,21^

Bile acid transporter genes belong to two super-families of transporters: ATP-binding cassette (ABC) and solute carrier (SLC) transporters that achieve an important role in the canalicular secretion and transport of bile and cholesterol in hepatocytes.^22^ The expression of these transporters is tightly regulated at the transcriptional and post-transcriptional levels.^23^ On the one hand, gut microbiota has been shown to play a critical role in bio-transformation of bile acids through deconjugation, dehydroxylation, and reconjugation processes.^21^ On the other hand, bile acids have antimicrobial activity which, in return, regulates the qualitative and quantitative composition of the microbiota and protects the epithelial barrier from damage.^24^ These functions are performed by primary or secondary bile acids, which are derived from bile salts.^25^ The bile acids have the ability to disrupt bacterial membranes, denature their proteins, and cause oxidative damage to DNA, thereby controls the gut microbiota species in the host.^26^

The liver coordinates the storage, breakdown and redistribution of nutrients efficiently to meet energy demands in different tissues. The hepatic energy metabolism is tightly regulated by different metabolic hormones, nutrients and neuronal signals.^27^ The liver also plays a central role in the regulation of bile acids metabolism.^28^ Variations in bile acid flow may initiate several types of liver diseases or impede the progression of an existing liver disease.^29^

Acute cholestasis is an acute liver injury due to an impairment of natural flux of bile acids from the liver to intestine due to an obstruction to the bile flow through intra/extrahepatic bile ducts. This results to an accumulation of cytotoxic bile acids in the liver and plasma and causes hepatocellular necrosis and apoptosis, acute liver inflammation followed by the proliferation of bile duct epithelial cell, activation of hepatic stellate cells and deposition of fibrotic tissue in the liver primarily as tissue repair and regenerative response.^30,31^ However, if the injury persists, the liver undergoes a sustained wound healing response where regeneration, inflammation and tissue remodeling occurs in a vicious circle leading to the formation of fibrotic scars and ultimately liver fibrosis.^32,33^

The impairment of bile flow to the intestine has also been reported to result in metabolic disorders modulated by the microbiota and associated with the occurrence of cholestasis.^34,35^ However, mechanisms underpinning the potential role of microbiota mediated pathogenesis and progression of cholestatic liver disease is not well understood. Therefore, in this study, we aimed to characterize the changes in hepatic gene expression profile and plasma BA composition modulated by varying microbial colonization under basal conditions and in acute cholestasis.

## Materials and Methods

### Animals

Male C57BL/6, aged 10–12 weeks, GF (germ-free) and ASF (altered Schaedler’s flora) mice were generated in Clean Mouse Facility (CMF) of University of Bern. The ASF mice contains eight bacterial species including *Lactobacillus acidophilus, Lactobacillus murinus, Bacteroides distasonis, Mucispirillum schaedleri, Eubacterium plexicaudatum*, a Fusiform-shaped bacterium and two *Clostridium* species and has the advantage to limit the possible experimental variability that can be expected with specific pathogen-free mice (SPF). Mice were born and maintained in flexible film isolators under HEPA air and fed with autoclaved chow and water ad libitum. All animals were kept on a 12-hour dark/light cycle. All experimental protocols obtained the approval of the Research Animal Ethics Committee of Canton Bern (BE66/10). All experiments were performed according to international guidelines concerning the conduct of animal experimentation.

### Induction of acute cholestasis by common bile duct ligation

As described previously,^36^ the mice received 60 µg/kg body weight of buprenorphine subcutaneously (Reckitt Benckiser, 0.3 mg/ml) before the surgery. Midline laparotomy was performed under isoflurane anesthesia. The bile duct was isolated from the surrounding tissues, and a 7–0 silk ligature was tied at proximal and distal end to the liver hilus. Finally, the bile duct was resected between the two ligatures. In sham-operated animals, the bile duct was only isolated but not ligated. The animals were sacrificed 5 days after BDL, plasma and tissues were collected for further experiments.

### Liver histology and image analysis

Segments of liver tissue samples were fixed in 4% buffered formalin, embedded in paraffin blocks and tissue slides were prepared for standard histology. The 5 µm sections were used for hematoxylin and eosin (H&E) staining for routine examination and quantification of bile infarcts as well as Sirius red staining for determination of fibrotic tissue in the liver. The quantification of bile infarcts was performed using MetaMorph imaging software (NX software 64-bite, version 7.8.12.0). The focal necrosis surface for the whole liver section area was evaluated blindly from different randomly chosen field of areas.

### Multiplex fluorescence immunostaining

In-depth cell phenotyping was performed with multiplex immunostaining as described elsewhere.^37^ Briefly sequential immunostaining and antibody stripping was performed on 4 µm-thick formalin-fixed paraffin embedded liver tissue sections obtained from all experimental groups (n = 3/group). After deparaffinization with xylene, all tissue sections underwent repetitive cycles of antigen retrieval (Tris-EDTA, pH 9.0, Novus Biologicals, Centennial, CO, USA) for 30 minutes in water bath and antibody stripping using 2-mercaptoethanol/SDS as described previously.^38^ To avoid nonspecific antibody binding the sections were incubated with 2.5% horse serum (Vector BioLabs, Malvern, PA, USA), for 1 h at room temperature for the first round of staining. Afterward, the sections were incubated overnight at 4°C with primary antibodies (Supplementary table 1S). Whole scanning of sections was performed using Zeiss Axio Observer 7 followed by stitching and background subtraction. The scans were aligned, hyperstacks and concatenated using FIJI HyperStackReg V5.6 plugin. The image segmentation was performed on all binary images using Ilastik software (v 1.3.3). CellProfiler v3.1.9 was used for cell identification, counting and intensity measurement.

### RNA extraction and mRNA sequencing

Total RNA was extracted from 30 to 50 mg of frozen liver tissues using the RNeasy Plus Mini Kit (Qiagen), as described in manufacturer’s instructions. RNA was eluted in 30 μl of RNase and DNase free water. The quantity and quality of the extracted RNA was assessed by checking the RNA integrity number (RIN) using the Agilent 2100 bioanalyzer. The concentration of RNA was evaluated using Agilent RNA 6000 Nano kit (5067–1512). Total RNA concentration of 1–2 µg was used for mRNA sequencing (Illumina Hiseq 2500). The NGS reads were mapped to the mouse reference genome mm10) with Tophat v.2.0.13.^39^ We used htseq-count v.0.6.1 to count the number of reads overlapping with each gene as specified in the Ensembl annotation release 75.^40^ The number of reads per gene were compared between the different experimental groups using DESeq2 v.1.6.3.^41^ *P*-values were FDR adjusted using the procedure of Benjamini and Hochberg 1995.^42^ All gene expression volcano plots were generated using ggplot package in R. Genes with an absolute log fold change > 2 and adj-*p* value < .05 were considered significant. Furthermore, known and predicted interactions for differentially regulated genes were derived using the STRING database.^43^

### Gene expression analysis by real-time quantitative PCR

Liver or small intestine sections of 30 to 40 mg were used to extract RNA with the RNeasy Plus Mini Kit (Qiagen) and total RNA was quantified using NanoDrop™ OneC (ThermoFisher). cDNA was obtained by reverse transcription of 0,15 µg of RNA using M-MLV Reverse Transcriptase (ThermoFisher) and oligo (dT) primers. qPCR reactions were performed using predesigned probes and primers (Supplementary table 1S) and TaqMan™ Fast Universal PCR Master Mix (ThermoFisher) in a QuantStudio™ 3 Real-Time PCR System (ThermoFisher) according to the manufacture’s protocol. C_T_ values were normalized to 18S ribosomal RNA housekeeping gene and represented as relative fold change to the corresponding control condition (2^−ΔΔCt^).

### Protein gel electrophoresis and Immunoblot Analysis

Frozen liver tissues were thawed and washed twice with ice-cold PBS. Livers were homogenized with 0.5 mm zirconium oxide beads (Next Advance, Troy, NY, USA) in a bullet blender (Next Advance, Troy, NY, USA) in RIPA buffer (150 mM NaCl, 1% NP-40, 0.5% Na-deoxycholate, 0.1% SDS, and 50 mM Tris-HCl, pH 7.4) containing Halt™ protease and phosphatase inhibitor (Thermo Fisher Scientific, Rockford, IL, USA). Protein concentration was measured with the Pierce^TM^ BCA assay (Thermo Fisher Scientific, Rockford, IL, USA). 15 µg of total lysate was loaded into 10–12% sodium dodecyl sulfate polyacrylamide gel and proteins were resolved by electrophoresis. The proteins were transferred to nitrocellulose membranes, blocked for 1 h with 5% nonfat milk, and then incubated overnight at 4°C with primary antibodies (Supplementary table 1S). Next, the membranes were incubated with peroxidase-conjugated secondary antibody (Thermo Fisher Scientific, Rockford, IL, USA) for 1 h and signals were revealed with enhanced chemiluminescence (Amersham ECL Prime, GE Healthcare, Glattburg, Switzerland) and a Fusion CCD camera coupled to a computer equipped with Fusion Capt Fx Software (Vilber-Lourmat, Marne-la-Vallée, France). Band densitometry was measured with the Bio-1D Advanced software (Vilber-Lourmat).

### Free fatty acids measurement

Ten milligrams of liver tissue was homogenized and total-free fatty acids were measured in 10 mg of liver tissue by the fluorometric FFA kit (BioVision) as per the manufactured instructions.

### Respirometry assay using modified Seahorse protocol

The oxygen consumption rates (OCRs) in homogenates from frozen livers were measured with a Seahorse Extracellular Flux (XF) ‐96 Bioanalyzer (Agilent Technologies, Santa Clara, CA, USA). Frozen liver tissues were thawed and washed two times with ice-cold PBS. The ‘Respirometry in Frozen Samples (RIFS)’ protocol was adapted from Acin-Perez R *et al*. 2020.^44^ Approximately, 50 mg of tissue was cut into small pieces and homogenized in MAS buffer (70 mM sucrose, 220 mM mannitol, 5 mM KH_2_PO_4_, 5 mM MgCl_2_, 1 mM EGTA, 2 mM HEPES, pH 7.4) with 20 strokes in a glass Dounce homogenizer. The homogenate was centrifuged at 1,000 g for 10 min at 4°C, the supernatant was collected and protein concentration was determined by Pierce^TM^ BCA assay (Thermo Fisher Scientific, Rockford, IL, USA). In Seahorse XF96 microplate, 10 µg (proteins) of homogenate was loaded into in 20 µl of MAS. The loaded plate was centrifuged at 2,000 g for 5 min at 4°C and an additional 130 µl of MAS containing cytochrome c (10 µg/ml) was added to each well. Substrates were delivered by port A (pyruvate + malate (5 mM each)) or NADH (1 mM), or 5 mM succinate + rotenone (5 mM + 2 µM). The inhibitors, Rotenone and antimycin A (2 µM + 4 µM) were delivered by port B. TMPD + ascorbic acid (0.5 mM + 1 mM) at port C; and azide (50 mM) at port D.

### Metabolomics

The blood samples were obtained from the inferior vena cava of sham and BDL mice both in GF and ASF colonized mice. The plasma was then extracted and frozen immediately at −80°C for longer storage. The plasma samples from all mentioned groups were used for metabolomics study as described below in Section *UHPLC-HRMS* Analyses.

### Bile acid extraction

Deuterated chenodeoxycholic acid (CDCA) and deoxycholic acid (DCA) were used as recovery standards in this study.^45^ 30 µl of the collected plasma (duplicates) from all experimental groups mixed with 10 µl of recovery standard solution (CDCA-D4 100 µM). Then, an amount of 100 µl of ice cold alkaline (5% NH_4_OH in ACN) was added and vortexed 1 min equally for all samples and continuously shaken for 1 hour at room temperature. The mixture was then centrifuged at 16,000 g for 10 minutes. The supernatant was collected and freeze-dried in a rotational vacuum concentrator overnight. Subsequently, the extracts were reconstituted in 80 µl of ammonium acetate (5 mM): methanol pH 6 and were kept in −20°C until LC-MS injection.

### UHPLC-HRMS Analyses

We performed qualitative and quantitative analyses of the plasma concentration of BA on an Agilent 6530 Accurate-Mass Q-TOF LC/MS mass spectrometer coupled to an Agilent 1290 series UHPLC system (Agilent Technologies, USA), as described elsewhere.^45^ Sample separation was performed using a Zorbax Eclipse-Plus C18 column (2.1 × 100 mm, 1.8 µm; Agilent Technologies, USA) heated at 50^°^C.5 mM ammonium acetate (pH 6) in water was used as eluent A and acetonitrile as eluent B in a binary gradient system. The detection in the mass spectrometer was operated in negative ionization mode using the Dual AJS Jet stream ESI Assembly. During acquisition the internal calibration was performed by infusing continuously the reference mass solution 5 mM purine, 1 mM HP-921 (Agilent reference mass kit, Agilent Technologies USA) in 95% MeOH acidified with 0.1% formic acid and allowed to permanently achieve a mass accuracy better than 5 ppm. HR mass spectra were acquired over the range of m/z 300–700 at an acquisition rate of three spectra/s. The data was processed using the Mass Hunter Workstation (Agilent Technologies, USA). Extracted ions chromatograms (XIC) were based on a retention time (RT) window of ±0.5 min with a mass extraction-windows (MEW) of ±30 ppm centered on ^m/z^_theor_ of each bile acid.

### Statistical analysis

Statistical analyses were performed using GraphPad Prism 5 (GraphPad Software Inc., California, USA). Comparisons between two groups were performed using Mann- Whitney’s U test. Multiple comparisons were performed using a one-way ANOVA followed by a Kruskal-Wallis test. Data are reported as mean ± SD. Differences were considered significant at <0.05. The FDR correction in NGS data was calculated according to Benjamini and Hochberg, 1995.^42^

## Results

### Absence of intestinal microbiota results in larger bile infarcts area and less ductular reactions in acute cholestasis

**Figure 1.**
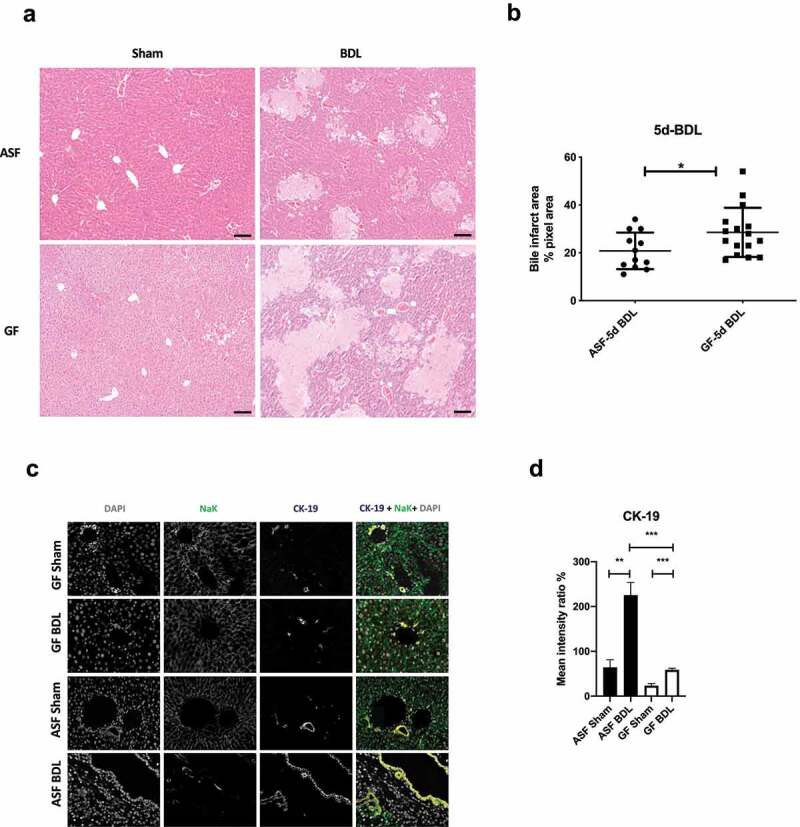
Acute cholestasis induce the formation of bile infarcts and ductular reactions in the liver of ASF and GF mice

To evaluate the extent of liver damage upon BDL, liver sections from experimental groups of mice were stained with hematoxylin and eosin (H&E). The intrahepatic accumulation of bile after BDL resulted in bile infarcts in both groups; GF-BDL and ASF-BDL mice (Figure 1a). However, the size of bile infarct areas was significantly greater in GF cholestatic mice as compared to ASF cholestatic mice (Figure 1b). To confirm the effect of cholestasis in GF and ASF-BDL mice, we performed multiplex immunostaining of CK-19 (Figure 1c). We observed a significant increase in CK-19 positive biliary cells in response to BDL in both GF and ASF mice as compared to their respective sham-operated mice. However, mice with microbiota showed more ductular reactions after acute cholestasis than mice without microbiota (Figure 1d).

(A) Representative H&E staining images at 10x magnification showing the formation of bile infarcts in liver sections of ASF and GF mice after 5 days of BDL. (B) Bile infarct area quantification (percentage pixel area) in liver sections of ASF and GF mice after 5 days of BDL. (C) Representative images of multiplex immunostaining of CK-19 in liver sections of all experimental groups and (D) quantification of positive CK-19. Data are expressed as mean ± SD. n=3/group. *p<0.05, **p<0.01, ***p<0.001. Abbreviations: ASF; altered Schaedler’s flora, GF; germ free, H&E; hematoxylin and eosin, CK-19; cytokeratin-19, SD; standard deviation. 

### Acute cholestasis differentially alters hepatic gene expression according to the presence or absence of intestinal microbiota

**Figure 2.**
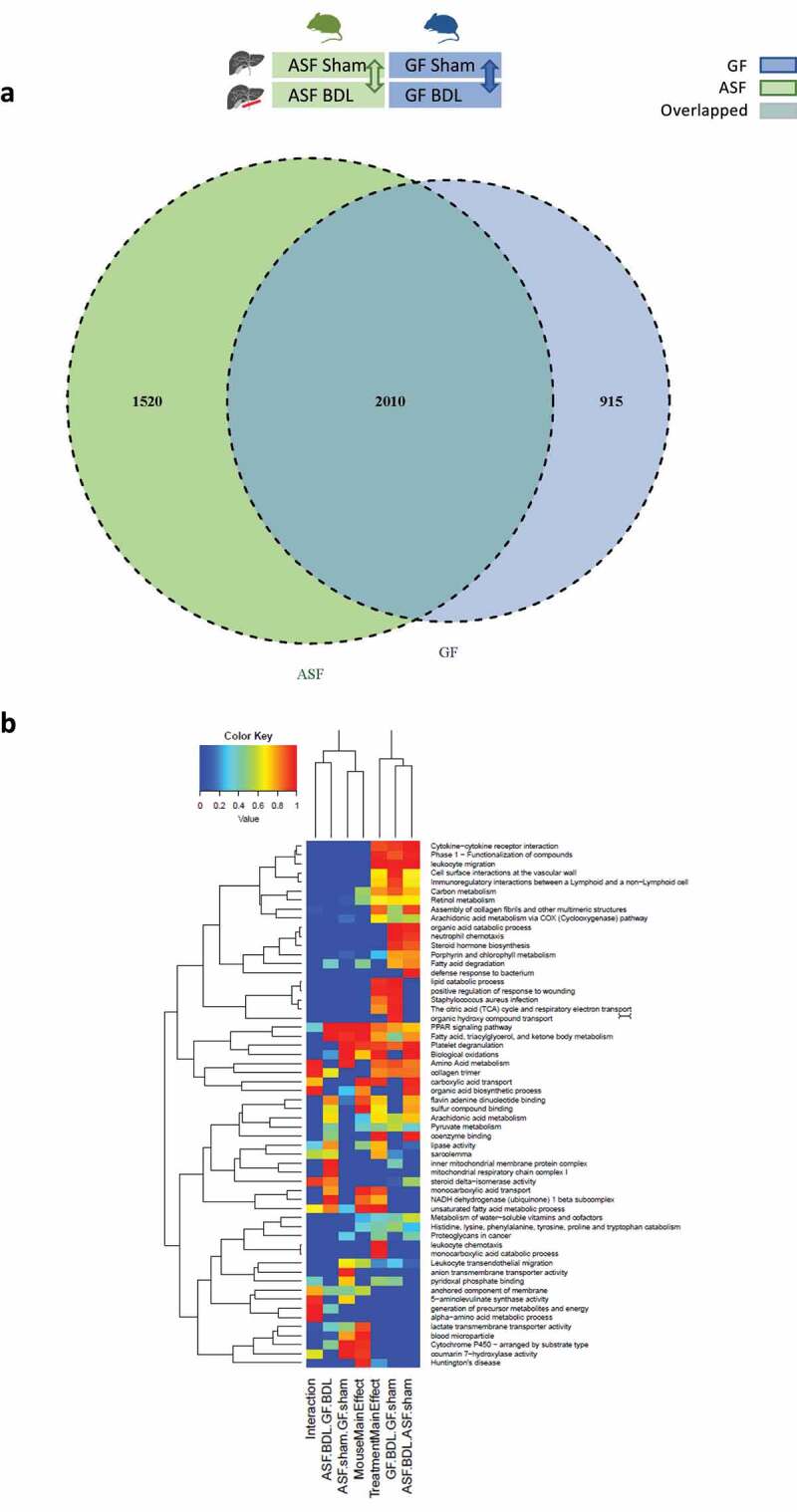
Transcriptomic analysis of liver samples from sham-operated or BDL mice with or without microbiota

To analyze the different response to acute cholestasis depending on hygiene status, hepatic gene expression patterns were evaluated by meta-transcriptomic analysis. The comparison was performed between the groups with distinct microbiota, in acute cholestasis and at baseline. Among the total of 4445 differentially expressed genes (DEGs), we observed 1520 genes were differentially expressed only in the comparison of ASF-BDL to ASF-sham, 915 genes only in the comparison of GF-BDL to GF-sham and 2010 genes in both overlapped between ASF-BDL and GF-BDL (Figure 2a). The Pathway enrichment analysis was further performed in different combinations across all experimental groups to find the functions of these DEGs (Figure 2b).

(A) Venn diagram showing the total number of DEGs after acute cholestasis in ASF and GF mice as compared to baseline. The figure also demonstrates the overlap of DEGs in GF and ASF after 5 days of BDL as compared to baseline. The DEGs with p<0.05 were considered significant, n=3/group. (B) Heat map showing an overview of differentially expressed gene sets with their associated biological pathways in GF vs ASF (hygiene status) before and after acute cholestasis. Blue color represents a lower and red color represents a higher expression level. Abbreviations: GF; germ free, ASF; altered Schaedler’s flora, BDL; bile duct ligation, DEG; differentially expressed genes.

### Absence of intestinal microbiota significantly alters hepatic gene expression at baseline

**Figure 3.**
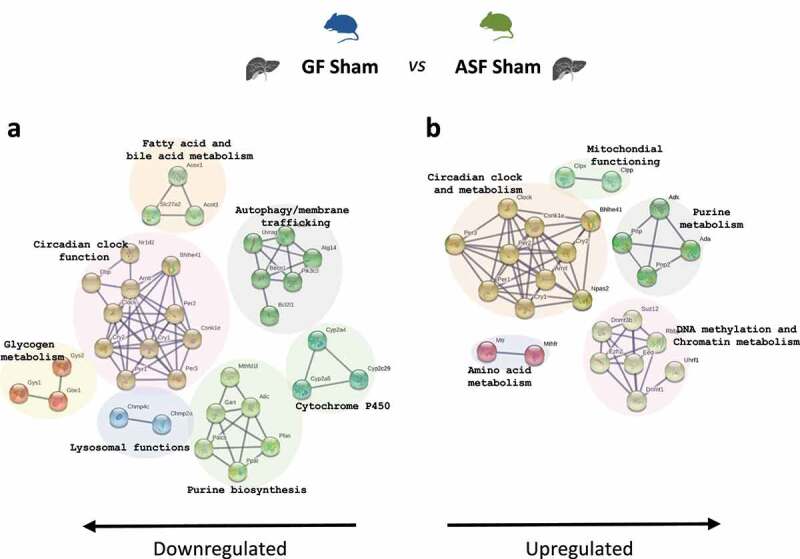
Transcriptome analysis of GF-sham vs ASF-sham

At baseline we found that 78 genes were differentially regulated when GF mice were compared to ASF mice (Supplementary figure 1S). Among those differentially regulated, 54 genes associated with the functions of fatty acid metabolism, circadian clock, autophagy, purine biosynthesis, cytochrome P450, lysosomal functions and glycogen metabolism were significantly downregulated at baseline in GF-sham *vs* ASF-sham (Figure 3a), and 24 genes associated with purine metabolism, circadian functions, DNA methylation, amino acid metabolism, and mitochondrial functions were significantly upregulated in GF-sham mice when compared to ASF-sham mice (Figure 3b). The details of the differentially regulated genes are shown in Supplementary table 2S.

(A and B) Pathway enrichment analysis of hepatic transcriptome data, Go enrichment analysis for biological processes among differentially regulated genes in ASF vs GF mice at baseline. p<0.05, n=3/group. Abbreviations: GO; gene ontology, ASF; altered Schaedler’s flora, GF; germ free. 

### Acute cholestasis significantly alters hepatic gene expression in the presence of microbiota associated with increased tissue repair response

**Figure 4.**
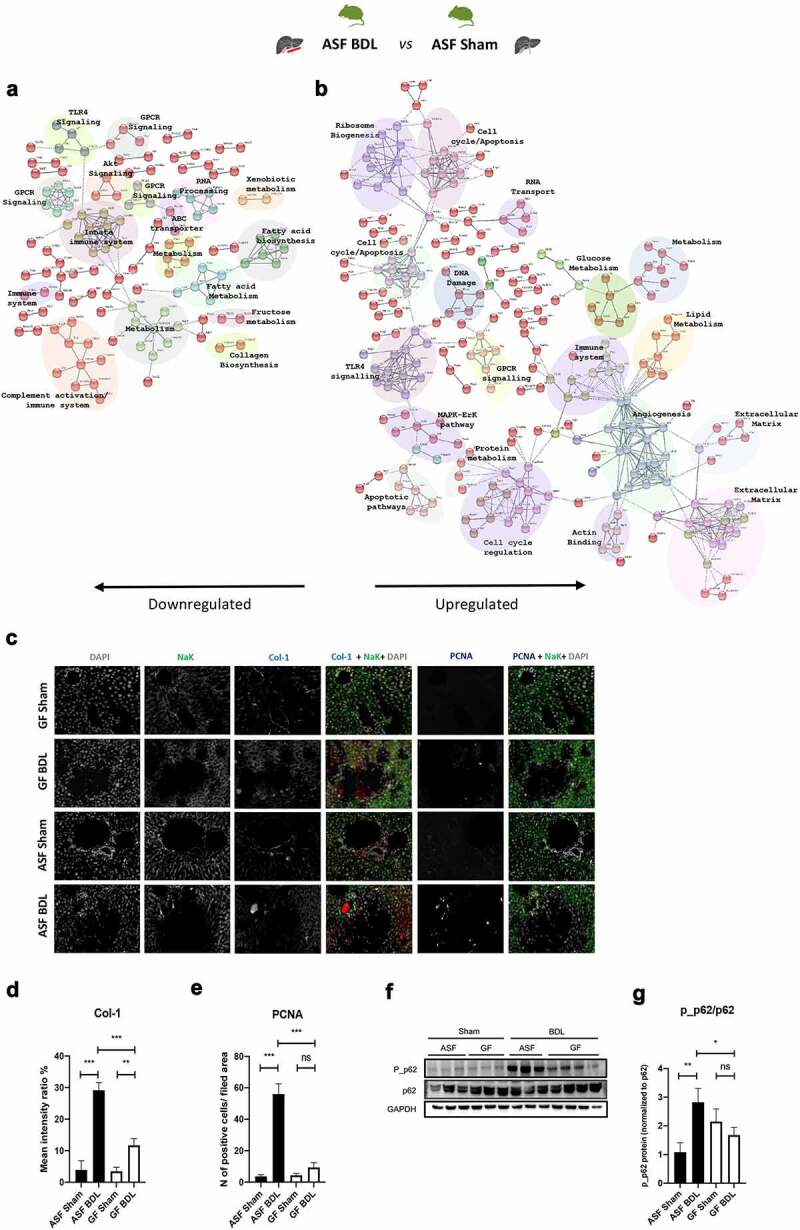
Transcriptome analysis of ASF-BDL vs ASF-sham reveals an increased regenerative response to the liver injury in mice with microbiota

We identified 1520 DEGs induced by acute cholestasis in ASF mice compared to controls. Among these 1520 genes, 716 genes were significantly downregulated, and 804 genes were significantly upregulated in ASF-BDL when compared to ASF-sham (Supplementary figure S2A). Pathway enrichment analysis revealed that the downregulated genes were mainly involved in the regulation of the innate immune system, complement activation, fatty acid biosynthesis/metabolism, GPCR signaling, AKT signaling, xenobiotic metabolism and ABC transporter expression (Figure 4a). The upregulated genes were mainly associated with extracellular matrix remodeling, angiogenesis, actin binding, lipid and glucose metabolism, MAPK-ERK pathway activation, DNA damage/apoptosis, and GPCR signaling (Figure 4b). The details of the differentially regulated genes are shown in Supplementary table 3S.

(A and B) Pathway enrichment analysis of transcriptome data, Go enrichment analysis for biological processes among differentially regulated genes in ASF-BDL vs ASF-sham. p<0.05, n=3/group. (C) Representative images of multiplex immunostaining of Col-1 and PCNA in liver sections of all experimental groups and (D-E) corresponding quantifications. (F) Immunoblots of p62 and p_p62 in liver homogenates from ASF and GF groups followed by (G) quantification. GAPDH served as a loading control. Data are expressed as mean ± SD. n=3/group. *p<0.05, **p<0.01, ***p<0.001. Abbreviations: GO; gene ontology, ASF; altered Schaedler’s flora, GF; germ free, BDL; bile duct ligation, Col-1; collagen 1, PCNA; proliferating cell nuclear antigen, p_p62; phosphorylated p62, GAPDH; glyceraldehyde 3-phosphate dehydrogenase; ns; not significant, SD; standard deviation.

Next, we evaluated the expression of genes involved in extracellular matrix (ECM), cell proliferation and autophagy pathways enriched in BDL conditions. We measured the expression of collagen-1 (Col1) and proliferating cell nuclear antigen (PCNA) by Multiplex immunostaining in liver sections to further validate NGS data (Figure 4c). We observed a significant increase in the expression of the these markers after acute cholestasis in both GF and ASF mice as compared to sham-operated animals. However, the expression of these genes found to be significantly lower in GF-BDL than ASF-BDL (Figure 4d-e). Increased deposition of hepatic collagen in response to acute cholestasis was further confirmed by Sirius red staining and the results were in line with RT-qPCR and NGS data that expression of Col1 increased after BDL but this increase was more in ASF-BDL as compared to GF-BDL (Supplementary figure S2B).

To evaluate the effect of acute cholestasis on autophagy, we examined p62-mediated autophagy by measuring levels of p62 and phosphorylated-p62 (p_p62) in liver homogenates by WB (figure 4f). The expression levels of p62 remains similar among all experimental groups (figure 4f) but the expression of p_p62 was significantly increased in ASF-BDL when compared to the ASF-sham and GF-BDL suggesting an activation of this pathway during acute cholestasis in the presence of microbiota (Figure 4g).

### Absence of microbiota significantly alters hepatic gene expression in response to acute cholestasis associated with increased hepatic inflammation

**Figure 5.**
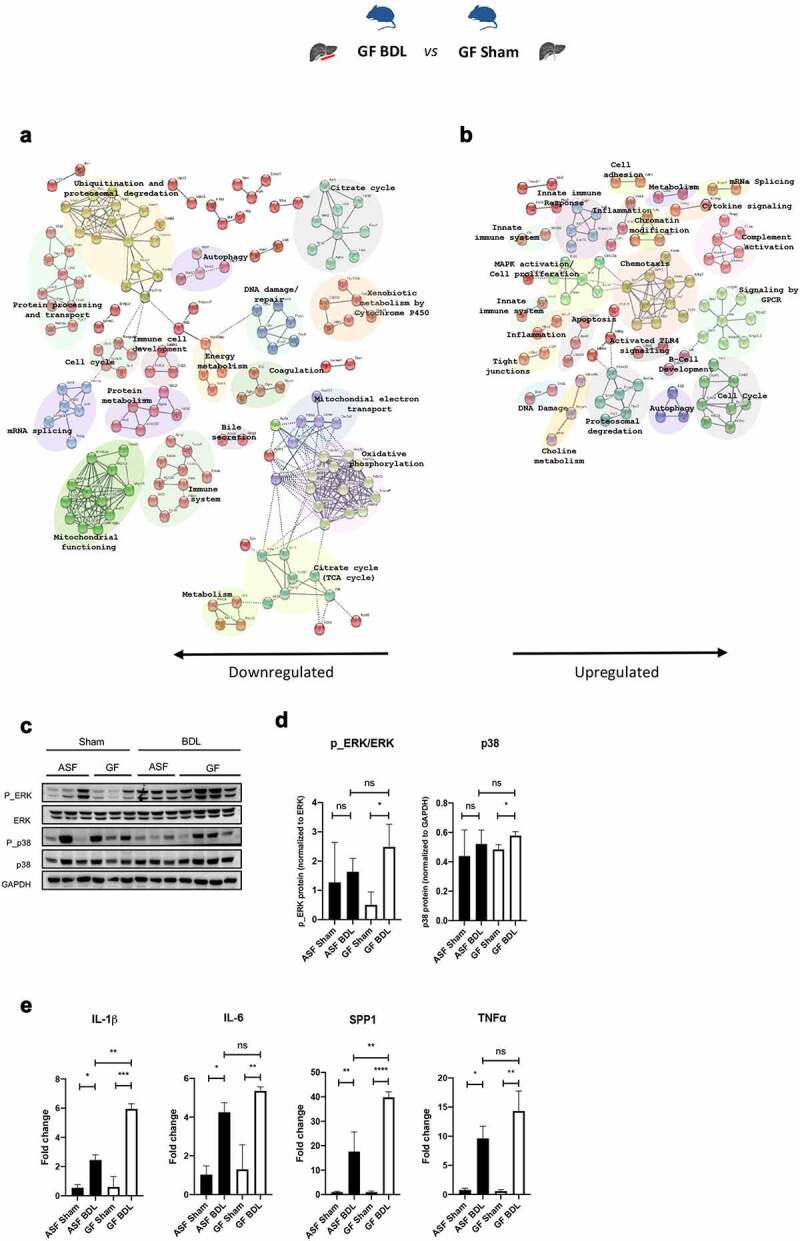
Transcriptome analysis of GF-BDL vs GF-sham shows increased hepatic inflammation after acute cholestasis in GF mice

Among the 915 differentially expressed genes, 464 genes were significantly downregulated, and 451 genes were significantly upregulated in GF-BDL when compared to GF-sham (Supplementary figure 3S). Pathway enrichment analysis revealed that the downregulated genes were mainly associated with energy/protein metabolism, xenobiotic metabolism by cytochrome P450, oxidative phosphorylation, bile secretion, immune cell development, DNA damage/autophagy, coagulation, protein processing and transport (Figure 5a). While the upregulated proteins were associated with the functions of chemotaxis, complement activation, inflammation, MAPK activation/cell proliferation, choline metabolism, apoptosis/autophagy, innate immune system, chromatin modification, and cell cycle (Figure 5b). The details of the differentially regulated genes are shown in Supplementary table 4S.

(A and B) Pathway enrichment analysis of transcriptome data, Go enrichment analysis for biological processes among differentially regulated genes in GF-BDL vs GF-sham. p<0.05, n=3/group. (C) Immunoblots of ERK and p38 and their phosphorylation in liver homogenates from all experimental groups followed by (D) corresponding quantification. GAPDH served as a loading control. (E) Liver proinflammatory markers including IL-1β, IL-6, SPP1 and TNF⍺ measured by RT-qPCR. Data are expressed as mean ± SD. n=3/group. *p<0.05, **p<0.01, ***p<0.001, **** p<0.0001. Abbreviations: GO; gene ontology, ASF; altered Schaedler’s flora, GF; germ free, BDL; bile duct ligation, ERK; extracellular signal-regulated kinase, p_ERK; phosphorylated ERK, GAPDH; glyceraldehyde 3-phosphate dehydrogenase, IL; interleukin, SPP1; osteopontin, TNF⍺; tumor necrosis factor, ns; not significant, SD; standard deviation.

In order to validate the data obtained from the pathways enrichment post BDL in the absence of microbiota, we first evaluated the activation of the MAPK signaling by measuring ERK, p38 and its phosphorylated form in liver homogenates by WB (Figure 5c). Phosphorylation of ERK and levels of p38 were significantly increased after acute cholestasis only when microbiota was absent (Figure 5d). We then assessed hepatic inflammation by measuring proinflammatory cytokines with RT-qPCR to confirm results obtained from NGS. Gene expression of proinflammatory markers (IL-1β, IL-6, SPP1, TNF-⍺) were significantly increased in response to acute cholestasis in the presence and absence of intestinal microbiota (Figure 5e). In line with MAPK activation, mRNA expression of IL-1β and SPP1 were significantly upregulated in the absence of microbiota when compared to ASF-BDL suggesting that mice without microbiota are more susceptible to liver inflammation after acute hepatic injury.

### Acute cholestasis alters hepatic gene expression associated with reduced lipid metabolism and mitochondrial respiration and gut microbes provide partial protection

**Figure 6.**
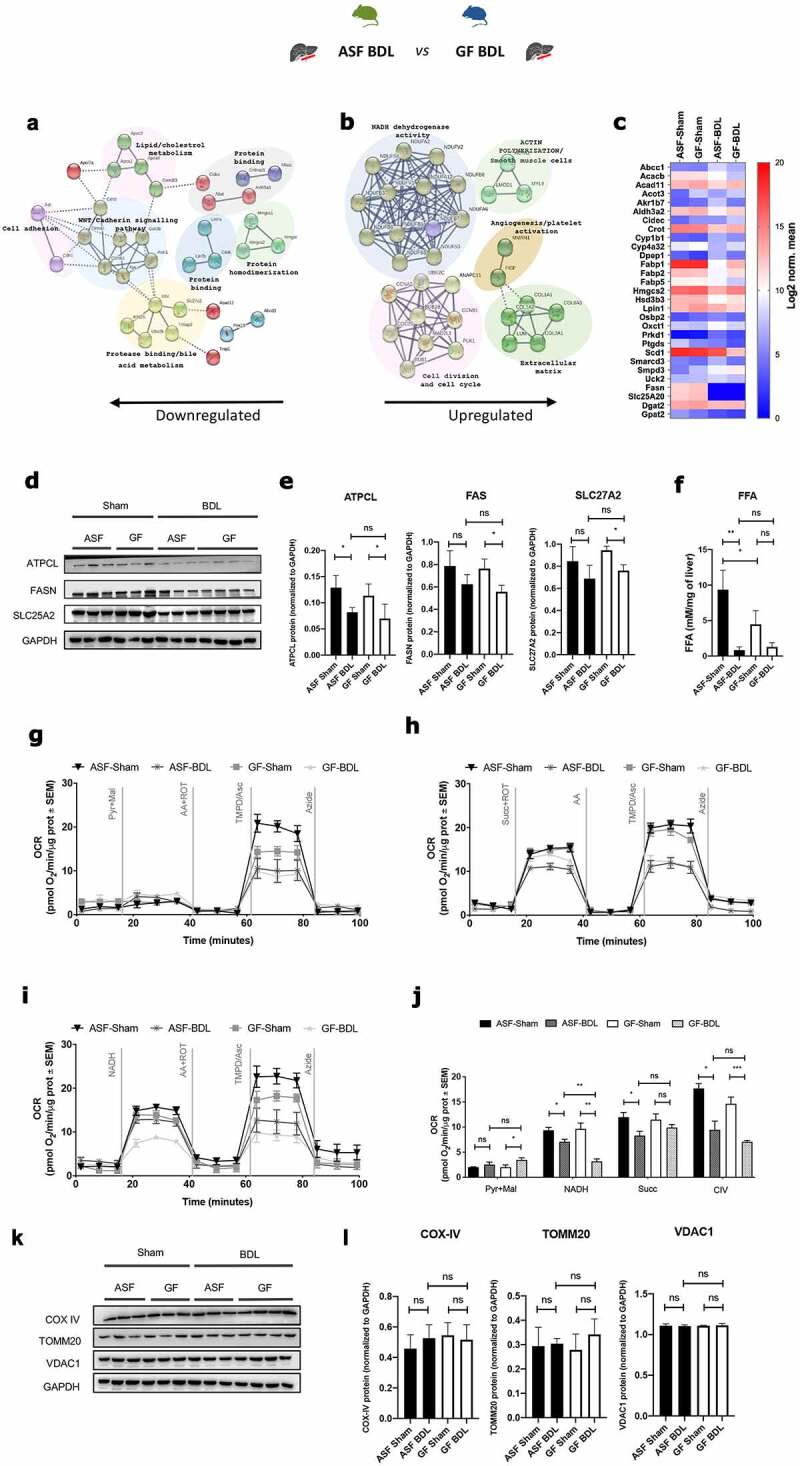
Transcriptome analysis of ASF-BDL vs GF-BDL shows that BDL affects lipid metabolism and mitochondrial functioning

We next compared only differentially expressed genes and their functional association (an effect of cholestasis) in ASF-BDL compared to GF-BDL mice. Among the 112 genes that were differentially expressed, 63 genes were significantly downregulated while 49 genes were significantly upregulated as shown in (Supplementary figure S4A). Pathway enrichment analysis showed that the downregulated genes were associated with the functions of lipid and cholesterol metabolism, protein binding/homodimerization, cell adhesion, protease binding, bile acid metabolism, and Wnt/Cadherin signaling (Figure 6a), while the upregulated genes were associated with processes such as NADH dehydrogenase activity, cell division/cell cycle, extracellular matrix, angiogenesis, platelet activation, and actin polymerization (Figure 6b). The details of the differentially regulated genes are shown in Supplementary table 5S.

(A and B) Pathway enrichment analysis of transcriptome data, Go enrichment analysis for biological processes among differentially regulated genes in ASF-BDL vs GF-BDL. p<0.05, n=3/group. (C) Gene expression of fatty acid metabolic genes significantly different between ASF-BDL and GF-BDL (p<0.05). (D) Immunoblots of fatty acid synthesis proteins in liver homogenates from all experimental groups followed by (E) quantification. GAPDH served as a loading control. (F) FFAs levels in liver homogenates from all groups included in the study. (G to J) Measurement of Complex I, II, and IV activity in liver homogenates in ASF and GF mice in BDL-induced acute cholestasis: Seahorse profiles of respirometry in the homogenates of frozen livers normalized to protein levels. (G) Pyruvate and malate used as substrates, (H) succinate used as a substrate, (I) NADH used as a substrate. (J) Quantification of the respirometry profiles of seahorse data. (K) Immunoblots of mitochondrial proteins in liver homogenates from ASF and GF groups followed by (L) quantification. GAPDH served as a loading control. Data are expressed as mean ± SD, or as mean ± SEM where indicated. n=3/group. *p<0.05, **p<0.01, ***p<0.001. Abbreviations: GO; gene ontology, ASF; altered Schaedler’s flora, GF; germ free, BDL; bile duct ligation, ATPCL; ATP citrate lyase, FAS; fatty acid synthase, SLC27A2; solute carrier family 27 member 2, GAPDH; glyceraldehyde 3-phosphate dehydrogenase, Pyr; pyruvate, Mal; malate, AA; antimycin A, ROT; rotenone, TMPD/Asc; TMPD + ascorbate, Succ; succinate, NADH; nicotinamide adenine dinucleotide hydrogen, OCR; oxygen consumption rate, SEM; standard error of mean, CIV; mitochondrial complex IV, COX; cytochrome c oxidase, TOMM; translocase of outer mitochondrial membrane, VDAC; voltage-dependent anion channel, ns; not significant, SD; standard deviation.

Fatty acid metabolism in the liver is impaired due to cholestasis.^46,47^ We also found enrichment of fatty acid metabolic pathways in our mRNA sequencing data while assessing the effect of BDL in liver (Figure 6c). Reduced expression of genes related to lipid metabolism after liver damage was validated by RT-qPCR (Supplementary figure S4B-D). We measured by WB the hepatic protein expression of ATP citrate lyase (ATPCL) that forms acetyl-CoA in cytosol as a precursor of lipogenesis, fatty acid synthase (FAS) that regulates the production of long-chain fatty acids from acetyl-CoA and the solute carrier family 27 member 2 (SLC27A2) that participates in the transport and uptake of fatty acids in the liver (Figure 6d). Expression of ATPCL was significantly reduced after BDL in both, ASF and GF mice. FAS and SLC27A2 expressions were also decreased after acute cholestasis but only significantly in animals without microbiota compared to sham-operated mice (Figure 6e). As expected, levels of free fatty acids (FFA) were reduced in both conditions after BDL but only signficanlty in ASF condition (figure 6f). Of note that levels of FFA at the baseline were significantly lower in the absence of microbiota underlining regulatory role of microbiota in lipid metabolism (figure 6f).

In mRNA sequencing data, we also found significantly enriched pathways related to mitochondrial bioenergetics while comparing ASF-BDL to GF-BDL (Figure 2b). Therefore, we measured mitochondrial mass and activities of its respiratory complexes (mitochondrial respiration complex I, complex II and complex IV activities) using Seahorse Extracellular Flux (XF) ‐96 Bioanalyzer in liver homogenates (Figure 6g-i). Our results demonstrated that in GF-BDL mice the pyruvate and malate-dependent basal respiration increased as compared to the GF-sham (Figure 6j). BDL in both groups, ASF and GF led to reduced activity of complex I but ASF-BDL had higher complex I activity than GF-BDL (Figure 6j). Further, the complex II activity was reduced in ASF-BDL compared to ASF-sham however; the absence of gut microbiota did not affect the complex II activity in sham or BDL groups (Figure 6j). Complex IV activity was measured independently, using the substrates TMPD/ascorbic acid and showed that BDL resulted to reduced activity of complex IV in both, ASF and GF mice compared to sham groups (Figure 6j). To determine whether the mitochondrial mass could be attributed to the reduction in the activity of respiratory complexes, we quantified mitochondrial proteins cytochrome c oxidase (COX) IV, translocase of outer mitochondrial membrane 20 (TOMM 20) and voltage-dependent anion channel 1 (VDAC1) in liver homogenates (Figure 6k). We found no difference in the expression of these proteins nor in ASF and GF shams neither in BDL groups indicating that the changes in the mitochondrial respiration are not due to the mitochondrial abundance but rather to its activity in our model of acute cholestasis (Figure 6l).

### Acute cholestasis results in increased concentration of primary BA independent of distinct microbiota

**Figure 7.**
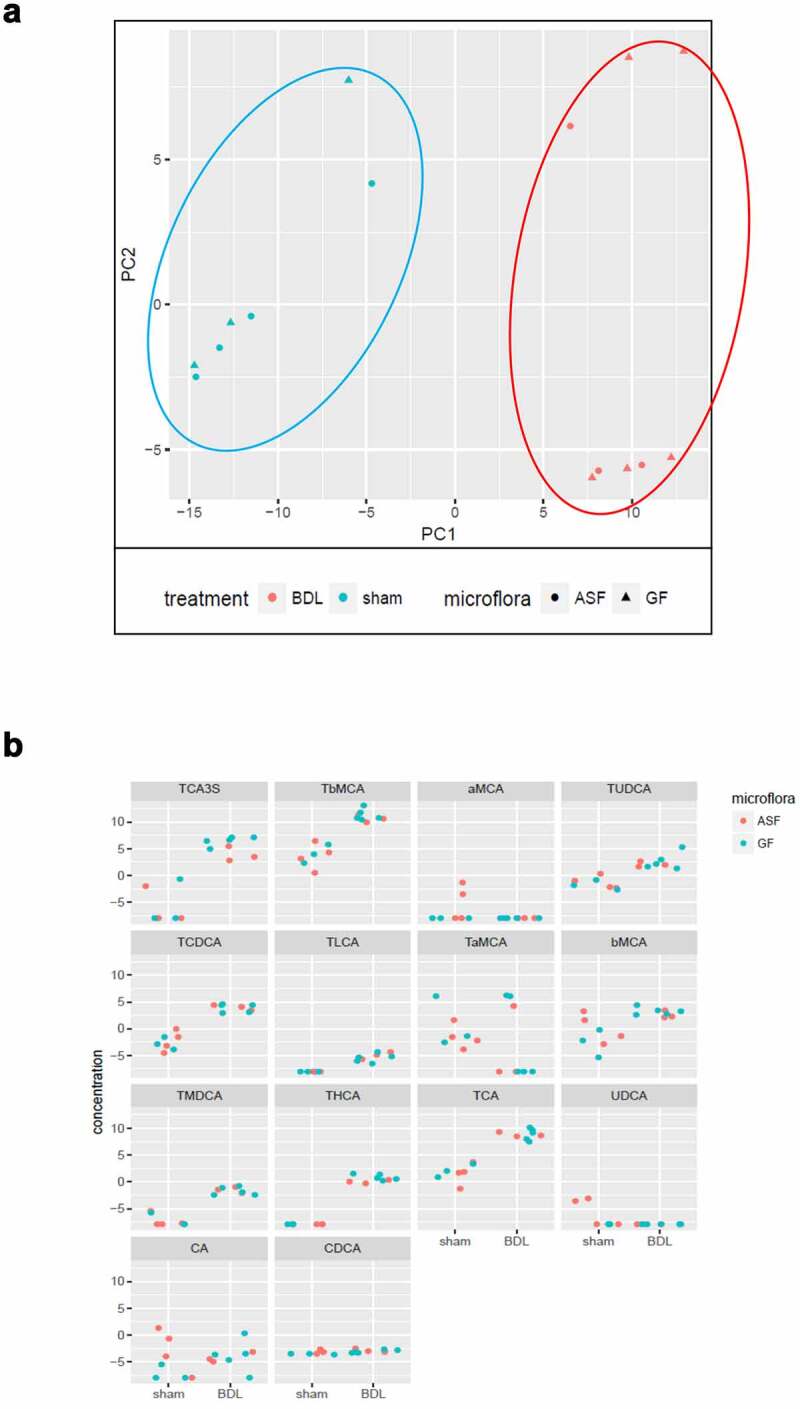
Analysis of plasma metabolomic profile in GF and ASF mice shows unremarkable differences before and after acute cholestasis

We next investigated whether the hygiene status and acute cholestasis affect the plasma metabolomic profile in mice. The principal component analysis is shown in Figure 7a. Under basal conditions, there were no significant differences in primary BA concentrations between GF and ASF mice. The concentration of the majority of BA markedly increased in acute cholestasis in both groups. However, we observed that the concentration of bMCA and tauro-conjugated primary bile acids like TCA3S, TbMCA, TUDCA, TCDCA, THCA, TCA were remarkably higher only in cholestasis without remarkable differences related to the hygiene status of the mice (Figure 7b).

Representative figures showing (A) Score blots showing PCA of plasma samples from sham (blue), with respect to BDL (red). (B) Bile acid profile in ASF vs GF after acute cholestasis. Abbreviations: ASF; altered Schaedler’s flora, GF; germ free, BDL; bile duct ligation PCA; principal component analysis.

## Discussion

In this study, we investigated the effects of distinct microbial colonization on hepatic gene expression in response to liver damage using an experimental model of acute cholestasis. We demonstrated here that gut microbes provide a partial protection to acute cholestasis induced by BDL associated with reduced regions of bile infarcts, increased regenerative/tissue repair response of the liver, balanced hepatic inflammation, and lipid metabolism as well as mitochondrial respiration while compared to GF mice. We have also identified by next generation sequencing sets of genes that were differentially expressed according to the presence or absence of intestinal microbiota, and with or without cholestasis.

Previously, *Mazagova et al* showed that the absence of microbiota was associated with increased liver fibrosis in chronic models of liver injury.^48^ In line with this data, *Tabibian et al* demonstrated severe biliary injury was associated with increased ductular reactions, fibrosis, ductopenia and cholangiocyte senescence in *mdr^−/-^* knockout mice under GF conditions.^49^ In a similar manner, we have also recently demonstrated a hepato-protective effect of complex microbiota in two different experimental models of liver disease.^36^ Mice lacking intestinal microbiota have more severe phenotype associated with metabolic functions, deficiencies in some of the vital vitamins and an immature immune system.^50,51^ Therefore, the results presented here reinforce the notion that in the absence of intestinal microbiota, acute cholestasis is associated with increased hepatic damage and metabolic dysfunction.

The increased susceptibility of GF mice to acute cholestasis was primarily evidenced when we observed that survival rate in GF-BDL mice drastically reduced (Supplementary Figure 5S) which further confirmed with larger regions of bile infarcts observed in GF compared to ASF-colonized mice. To investigate whether differences in intestinal microbial colonization during acute cholestasis may influence the expression of hepatic genes, we studied gene expression profiles in liver samples from GF and ASF mice at baseline and in acute cholestasis. We observed differentially expressed gene sets involved mainly in ECM, cell cycle, autophagy, activation of MAPK, inflammation, metabolism of lipids and mitochondrial functioning pathways being differentially affected according to the mice hygiene status. We therefore characterized our model and investigated the most relevant pathways in details. We found that mice were more responsive to acute liver injury in the presence of microbiota as evidenced by increased ductular structures, hepatocyte proliferation, deposition of Col-1 and activated autophagy indicating an increased hepatic regenerative response 5 days after acute cholestatic. Ductular reactions in early stages after injury reflect liver regenerative response to compensate loss of biliary and hepatocyte cell populations.^52^ Increased DNA synthesis detected by staining of PCNA in ASF-BDL represents increased hepatocyte proliferation associated with greater liver regeneration in the presence of microbiota. Similarly, enhanced expression of Col-1 and p_p62 (autophagy) observed in ASF-BDL are consistent with active regeneration and tissue repair process thereby providing hepatoprotective response during acute liver injury.^30,53^ We therefore concluded that the presence of gut microbiota is associated with early physiological wound healing and tissue repair responses which are considerably impaired in GF mice during acute cholestasis.

As expected, hepatic inflammation was significantly increased after BDL in the presence and absence of microbiota. However, we found more inflammation and immune responses pathways being altered in GF-BDL such as ERK/MAPK signaling pathway. We further validated these findings by evaluation of gene expression levels of proinflammatory cytokines such as TNF-α, IL-1β, IL-6 and SPP1 which were significantly upregulated in both groups but this increase was more in GF-BDL mice as compared to ASF-BDL. Moreover, the exacerbated inflammatory reponses observed in GF mice may correlate to their immature and under-developed immune system associated with defective number and function of regulatory T cells controlling proinflammatory responses.^54,55^ The overexpression of SPP1 observed in GF-BDL can mediate the aggravated liver injury by recruitment of neutrophils to the damaged area thereby induces necrosis by attacking stressed cells.^32^ GF mice susceptibility to aggravated inflammatory responses after BDL predispose them to extended acute liver injury, further supporting the role of gut microbes in alleviating liver injury.

As observed in NGS data, the expression of genes related to lipid metabolism and functioning of mitochondria were affected in our model. We further confirmed reduced expression of lipid metabolism-related genes; fatty acid-binding protein (Fabp)-1, fatty acid synthase (Fasn) and peroxisome proliferator-activated receptor (Ppar)-⍺ and ATPCL protein in response to hepatic damage in all experimental groups. However, the lipid metabolism found to be more affected in GF mice as they showed reduced protein expression of FAS and SLC27A2 after BDL and importantly reduced levels of FFA at baseline when compared to ASF-sham. Additionally, we evaluated the activity of the mitochondrial respiratory complexes I, II and IV to assess the capacity of mitochondria to produce ATP through oxidative phosphorylation. We found that the mitochondrial activity was affected more in the absence of microbiota after BDL. These results further support that energy supplies in GF mice are altered considerably early during liver injury and it might be associated with an impaired capacity of the liver to regenerate as reported elsewhere.^56^ The impact of the gut microbiota in the regulation of metabolic functions is still poorly understood. *Bäckhed et al* reported that in the absence of gut microbiota, mice have less hepatic fatty acids and adipose triglycerides compared to conventionally-raised mice.^57,58^ On the other hand, *Kimura et al* have reported that intestinal bacteria can suppress hepatic fatty acid synthesis and reduce the accumulation of lipids in adipose tissue.^59^ Our findings are in line with the study of *Bäckhed* and colleagues suggesting that hepatic metabolic functions are controlled by gut microbiota.^57,58^ The changes in microbiota composition could affect the occurrence and development of liver disease^34,60^ . The observations by *Kindt et al* also demonstrated that intestinal microbiota promote fatty acid desaturation and elongation, therefore supporting its regulatory role in lipid metabolism.^14^ These data are in line with our findings that hepatic fatty acid metabolism is critically impaired in GF mice due to the absence of intestinal microbiota.

Bile acids are synthesized from cholesterol in the liver and gut microbiota plays an important role in bile acid metabolism and recirculation.^20,61^ We investigated whether hygiene status or acute cholestasis affects the plasma bile acid profile in mice. The concentration of free and conjugated bile acids (BA profile) in plasma samples from GF and ASF colonized mice at baseline and after cholestasis was evaluated. We did not find significant differences in concentration of primary bile acid profile under basal conditions between GF and ASF mice. Nevertheless, we observed an increased concentration of bile acid only after acute cholestasis independent of mice hygiene status. Limited colonization did not show any remarkable alteration in plasma bile acid concentrations as compared to GF mice. Therefore, we acknowledge that limited colonization (far from conventionally raised mice) may not be sufficient to significantly influence the serum bile acid profile in ASF mice in comparison to GF mice. Hence, this may partly explain the unremarkable differences of bile acid profile between ASF and GF mice.^62^

In summary, we demonstrated here that complete absence of intestinal microbiota contributed to the severity of hepatic injury during acute cholestasis. Liver injury in GF mice was associated with an increased inflammatory responses and impaired fat and energy metabolism leading to insufficient energy supply and therefore impaired hepatic regeneration. Alterations observed after BDL suggest that microbial-induced differences may affect the course of cholestasis and modulate liver injury. These findings highlight the beneficial role of gut microbiota for the host health as well as in the early stage of acute cholestasis and suggest that modifications of the gut microbiota may be beneficial in attenuating liver injury induced by cholestasis. One possibility to evaluate the beneficial role of microbiota in acute cholestasis is to transplant ASF microbiota into GF-BDL mice. This may lead to reduced hepatic damage, inflammation and improved fat and energy metabolisms in GF-BDL mice that needs to be investigated.

## Supplementary Material

Supplemental Material

## Figures and Tables

**Table:** 

ASF	(Altered Schaedler’s flora)
Germ-free	(GF)
BDL	(Bile duct ligation)
BA	(Bile acids)
DEGs	(Differentially Expressed Genes)
Col1	(Collagen-1)
ECM	(Extracellular matrix)
p_p62	(Phosphorylated-p62)
FFA	(Free fatty acids)
PCNA	(Proliferating cell nuclear antigen)
ATPCL	(ATP citrate lyase)
OCRs	(Oxygen consumption rates)
COX	(Cytochrome c oxidase)
TOMM 20	(Translocase of outer mitochondrial membrane 20)
VDAC1	(Voltage dependent anion channel 1)
ATP-binding cassette	(ABC)
SLC27A2	(Solute carrier family 27 member 2)
CK-19	(Cytokeratin-19).
