# Supplementary material for: Intestinal microbiota drives cholestasis-induced specific hepatic gene expression patterns
Source: Gut Microbes. 2021 Apr 13;13(1):1911534. doi: 10.1080/19490976.2021.1911534 (PMC8049203; doi:10.1080/19490976.2021.1911534)
Supplement: Supplemental Material [file KGMI_A_1911534_SM0051.zip › Supplementary information/LEGENDS_TO_SUPPLEMENTARY_FIGURES.docx]

**LEGENDS TO SUPPLEMENTARY FIGURES**

**Supplementary figure 1.**

(A) Volcano plots showing differentially regulated (upregulated and downregulated) genes in GF-sham vs ASF-sham comparison. The red dots signify log2-fold change>2; p<0.05), n=3/group. Abbreviations: GF; germ free, ASF; altered Schaedler’s flora.

**Supplementary figure 2.**

(A) Volcano plots showing differentially regulated (upregulated and downregulated) genes in ASF-BDL vs ASF-sham comparison. The red dots signify log2-fold change>2; p<0.05), n=3/group. (B) Representative Sirius red staining in liver sections from all experimental groups. Abbreviations: ASF; altered Schaedler’s flora, GF; germ free, BDL; bile duct ligation.

**Supplementary figure 3.**

(A) Volcano plots showing differentially regulated (upregulated and downregulated) genes in GF-BDL vs GF-sham comparison. The red dots signify log2-fold change>2; p<0.05, n=3/group. Abbreviations: GF; germ free, BDL; bile duct ligation.

**Supplementary figure 4.**

(A) Volcano plots showing differentially regulated (upregulated and downregulated) genes in ASF-BDL vs GF-BDL comparison. The red dots signify log2-fold change>2; p<0.05, n=3/group. Gene expression levels of (B) Fabp1, (C) Fasn and (D) Ppar⍺ genes in the liver of all experimental groups. Data are expressed as mean ± SD. n=3/group. *p<0.05. Abbreviations: ASF; altered Schaedler’s flora, GF; germ free, BDL; bile duct ligation. Fabp1; fatty acid binding protein 1, Fasn; fatty acid synthase, Ppar⍺; proliferator-activated receptor ⍺.

**Supplementary figure 5.**

(A) Survival curve in sham-operated and ASF-BDL mice showing up to 50% survival rate14 day after BDL. (B) Survival curve in sham-operated and GF-BDL mice showing less than 50% survival rate only up to 5 days after BDL.
