# Supplementary material for: Intestinal microbiota drives cholestasis-induced specific hepatic gene expression patterns
Source: Gut Microbes. 2021 Apr 13;13(1):1911534. doi: 10.1080/19490976.2021.1911534 (PMC8049203; doi:10.1080/19490976.2021.1911534)
Supplement: Supplemental Material [file KGMI_A_1911534_SM0051.zip › Supplementary information/Supplementary_table_1.docx]

**Supplementary table 1:** Details of antibodies used for multiplex staining.

| **S.No.** | **Reagent** | **Dilution** | **Source** | **Identifier** |
| --- | --- | --- | --- | --- |
| 1 | Rat anti-CK19 | 1:300 | Developmental Studies Hybridoma Bank | TROMA-III |
| 2 | Mouse anti-p62 | 1:300 | Abcam | Ab56416 |
| 3 | Rabbit anti-collagen1 | 1:500 | Abcam | Ab34710 |
| 4 | Mouse anti-PCNA | 1:1000 | Abcam | Ab29 |
| 5 | Anti-NaK ATPase | 1:400 | Abcam | Ab254025 |

**Supplementary table 2:** Details of probes used for TaqMan^Ⓡ^ gene expression assays.

| **S.No.** | **Reagent** | **Dilution** | **Source** | **Identifier** |
| --- | --- | --- | --- | --- |
| 1 | IL-1β | 1:20 | ThermoFisher Scientific | Mm00434228_m1 |
| 2 | IL-6 | 1:20 | ThermoFisher Scientific | Mm00446190_m1 |
| 3 | SPP1 | 1:20 | ThermoFisher Scientific | Mm00436767_m1 |
| 4 | TNF-⍺ | 1:20 | ThermoFisher Scientific | Mm00443258_m1 |
| 5 | IL-10 | 1:20 | ThermoFisher Scientific | Mm01288386_m1 |
| 6 | Fabp1 | 1:20 | ThermoFisher Scientific | Mm00444340_m1 |
| 7 | Fasn | 1:20 | ThermoFisher Scientific | Mm00662319_m1 |
| 8 | Ppar-⍺ | 1:20 | ThermoFisher Scientific | Mm00440939_m1 |
| 9 | Claudin-1 | 1:20 | ThermoFisher Scientific | Mm00516701_m1 |
| 10 | Occludin | 1:20 | ThermoFisher Scientific | Mm01349279_m1 |
| 11 | 18S | 1:20 | ThermoFisher Scientific | Hs99999901_s1 |

**Supplementary table 3:** Details of antibodies used for immunoblotting.

| **S.No.** | **Reagent** | **Dilution** | **Source** | **Identifier** |
| --- | --- | --- | --- | --- |
| 1 | Rabbit anti-phospho-AKT | 1:1,000 | Cell Signaling Technology | 9271 |
| 2 | Rabbit anti-AKT | 1:1,000 | Cell Signaling Technology | 9272 |
| 3 | Rabbit anti-phospho-mTOR | 1:1,000 | Cell Signaling Technology | 2971 |
| 4 | Rabbit anti-mTOR | 1:1,000 | Cell Signaling Technology | 2983 |
| 5 | Rabbit anti-phospho-ERK | 1:1,000 | Cell Signaling Technology | 4370 |
| 6 | Rabbit anti-ERK | 1:1,000 | Cell Signaling Technology | 9102 |
| 7 | Rabbit anti-phospho-p38 MAPK | 1:1,000 | Cell Signaling Technology | 4631 |
| 8 | Rabbit anti-p38 MAPK | 1:1,000 | Cell Signaling Technology | 9212 |
| 9 | Rabbit anti-GAPDH | 1:1,000 | Cell Signaling Technology | 2118 |
| 10 | Rabbit anti-phospho-p62 | 1:1,000 | Cell Signaling Technology | 13121 |
| 11 | Rabbit anti-p62 | 1:10,000 | Abcam | Ab109012 |
| 12 | Rabbit anti-ATPCL | 1:1,000 | Cell Signaling Technology | 4332 |
| 13 | Rabbit anti-FAS | 1:1,000 | Cell Signaling Technology | 3180 |
| 14 | Rabbit anti- SLC27A2 | 1:1,000 | Proteintech | 14048-1-AP |
| 15 | Rabbit anti-COXIV | 1:1,000 | Proteintech | 11242-1-AP |
| 16 | Rabbit anti-TOM20 | 1:1,000 | Abcam | Ab186734 |
| 17 | Mouse anti-VDAC1 | 1:1,000 | Abcam | Ab14734 |
